# High endogenous expression of parathyroid hormone-related protein (PTHrP) supports osteogenic differentiation in human dental follicle cells

**DOI:** 10.1007/s00418-020-01904-7

**Published:** 2020-07-24

**Authors:** Oliver Pieles, Anja Reck, Christian Morsczeck

**Affiliations:** Department of Oral and Maxillofacial Surgery, University Hospital Regensburg, Franz-Josef-Strauss-Allee 11, 93053 Regensburg, Germany

**Keywords:** Dental follicle, Stem cells, Osteogenic differentiation, PTHrP

## Abstract

Dental follicle cells (DFCs) are progenitor cells for mineralizing cells such as alveolar osteoblasts, but little is known about the mechanisms of the differentiation. Interestingly, different cell lines sometimes have different potentials to differentiate into mineralizing cells. In this study, we compared two different DFC lines, with one cell line (DFC_B) showing a high alkaline phosphatase (ALP) activity in long-term cultures with standard medium and a reliable mineralizing potential. However, the other cell line DFC_A shows low ALP activity in standard medium and almost no mineralization. Known osteogenic markers such as RUNX2 were similarly expressed in both cell lines. However, the proosteogenic signaling pathway of the bone morphogenetic protein (BMP) is induced in DFC_B, and the parathyroid hormone-related protein (PTHrP), which is involved in tooth root development, was also expressed more strongly. Previous studies have shown that the secreted PTHrP negatively regulate the transition from pre-osteoblastic progenitors to osteoblasts, but we showed that an inhibition of PTHrP gene expression reduced the ALP activity and the BMP-signaling pathway. In addition, endogenously expressed PTHrP is located in the cell nucleus. In contrast, supplementation of PTHrP or an inhibitor for the PTHrP receptor did not affect the ALP activity of DFC_B. In conclusion, our data suggest that a high endogenous expression of PTHrP in DFCs supports the induction of osteogenic differentiation via an intracrine mode.

**Electronic supplementary material:**

The online version of this article (10.1007/s00418-020-01904-7) contains supplementary material, which is available to authorized users.

## Introduction

Dental stem cells such as dental follicle cells (DFCs) could be used in the future to cure oral diseases (Morsczeck and Reichert 2018; Zhang et al. 2019a; Zhou et al. 2019) and were already successfully used for the regeneration of craniofacial bone in numerous animal studies and case reports in humans (Sybil et al. 2020). Another application field is tissue engineering of dental tissues such as a complete tooth root for a novel type of dental implants (Sonoyama et al. 2006). Reliable protocols for the differentiation of stem cells are required to avoid complications, but little is known about the molecular processes that direct the differentiation of dental stem cells into cementoblasts or alveolar osteoblasts (Morsczeck 2015; Morsczeck and Reichert 2017). We previously identified transcription factors such as DLX3 (Morsczeck 2006) and extracellular factors such as the parathyroid hormone-related protein PTHrP (Klingelhöffer et al. 2016), which is crucial for the development of bone and tooth (Vortkamp 2000). PTHrP is expressed in the tooth germ and a recent article describes that a great amount of PTHrP-expressing DFCs become tooth root cells such as periodontal ligament cells, alveolar osteoblasts and cementoblasts (Nagata et al. 2020; Takahashi et al. 2019). The expression of PTHrP can be correlated with the development of tooth root cells. Takahashi et al. showed that the autocrine PTH/PTHrP receptor (PPR) pathway -mediated cell fate of DFCs is required for correct tooth root development. The PPR is also described as a link between stem/progenitor cells and the vascular niche in the bone microenvironment (Schiano et al. 2019). For example, extracellular PTH was shown to modulate mesenchymal stem cells (MSCs) and increase their proliferation rate while inhibiting senescence induction (Di Bernardo et al. 2010). Thus, PTHrP-secreting cells should be considered as possible regulators of stem/progenitor cell fate.

A previous study evaluated the influence of PTHrP on the osteogenic differentiation of DFCs under in vitro conditions (Klingelhöffer et al. 2016). DFCs secrete PTHrP protein during the osteogenic differentiation, but a supplementation of the osteogenic differentiation medium with PTHrP protein inhibited the alkaline phosphatase (ALP) activity, which is an important marker of the osteogenic differentiation. Interestingly, it was shown that depletion of PTHrP gene expression did not support DFC differentiation and did not regulate the hedgehog signaling pathway after induction of osteogenic differentiation in DFCs (Klingelhöffer et al. 2016). For dermal cranial bone development, it is generally believed that both Indian hedgehog (IHH) and PTHrP negatively regulate the transition from pre-osteoblastic progenitors to osteoblasts (Abzhanov et al. 2007).

However, our new study shed new light on the role of PTHrP for the osteogenic differentiation of DFCs. In this study, we compared two cell lines with different osteogenic differentiation potentials and different gene expression levels of PTHrP. Our new data suggest that the expression of PTHrP may support the osteogenic differentiation.

## Materials and methods

### Cell culture and cell transfection

Human dental follicle cells (DFCs), which were purchased from AllCells, were cultivated in DMEM (Dulbecco’s modified Eagle medium) supplemented with 10% fetal bovine serum and penicillin/streptomycin (all purchased from Sigma) as described previously (Morsczeck et al. 2019a). DFCs between cell passages 6 and 9 were used in this study.

For gene expression silencing, DFCs were transfected with specific siRNAs for PTHrP (purchased from Qiagen). The transfection protocol has been described before (Morsczeck et al. 2019b).

Proliferation of cells was measured with Cell Counting-Kit 8 (CCK8, purchased from Dojindo) according to the manufacturer’s instructions.

### Osteogenic differentiation

After reaching sub-confluence (> 80%), DFCs were cultivated in the osteogenic differentiation medium (ODM) which is standard cell culture medium supplemented with 20 mM HEPES, 10 mM β-glycerophosphate, 100 µM phospho-ascorbic acid and 100 nM dexamethasone (all Sigma). Alternatively, the StemPro™ Osteogenesis Differentiation Kit (Thermo Scientific, #A1007201) was used for the induction of differentiation.

The osteogenic differentiation of DFCs was determined by alkaline phosphatase (ALP) activity. For this, DFCs were washed with PBS buffer, lysed in 0.1% Triton X-100 (in PBS) and incubated in alkaline phosphate yellow liquid substrate (Sigma) at 37 °C for 60 min, before the reaction was stopped by adding 3 M NaOH. The amount of liberated p-nitrophenol was measured spectrophotometrically at 405 nm. ALP activity values were normalized to total protein concentrations determined by BCA protein assay (Thermo Scientific).

The assessment of mineralization was analyzed after 4 weeks of osteogenic differentiation by Alizarin Red staining. The staining procedure has been previously described (Morsczeck et al. 2005).

To assess the influence of extracellular PTHrP on the alkaline phosphatase activity, DFCs were cultivated in the presence of 500 ng/ml PTHrP (PreproTech) and 350 ng/ml PTH (7–34) (Promokine) in ODM.

### Western blot analyses

Soluble cytoplasmic proteins were isolated from DFCs with a specific protein isolation buffer (20 mM Tris/HCl pH 8.0, 137 mM NaCl, 48 mM NaF, 2 mM Na_3_VO_4_, 1% NP-40, 10% glycerol, 1% phosphatase inhibitor cocktail 3 from Sigma, 1 cOmplete™ mini protease inhibitor cocktail tablet per 10 ml from Sigma). Samples were separated by SDS–polyacrylamide gel electrophoresis in 4–15% Mini PROTEAN^®^ TGX Stain-Free™ Protein Gels (BIO-RAD) which were then activated with UV light before blotting the proteins to a nitrocellulose membrane as described before (Morsczeck et al. 2019b). Images of total lane protein were taken. Primary antibodies for SMAD 1 (Cell Signaling #6944) and SMAD 1/5 phosphorylated at Ser463/465 (pSMAD, Cell Signaling #9516) and HRP-linked secondary antibodies were used according to the manufacturers’ instructions (Cell Signaling). The detection of antibody-marked protein bands was performed by chemiluminescence signaling developed with Clarity Western ECL Substrate (BIO-RAD) and measured with the ChemiDoc Touch Imaging System (BIO-RAD). Total lane protein was used as the loading control.

### Real-time reverse-transcription (RT) PCR gene expression analyses

For the evaluation of gene expression, total RNAs from DFCs were isolated and reverse transcribed as described previously (Prateeptongkum et al. 2016). PCR primer mixes for specific osteogenic marker genes were purchased from BIO-RAD (PrimePCR). The standard protocol for BIO-RAD PrimePCR primers was used with SsoAdvanced™ Universal SYBR^®^ Green Supermix (BIO-RAD) on the StepOnePlus real-time PCR machine (Applied Biosystems). The relative gene expression was calculated with the ΔΔC_t_-method (Winer et al. 1999).

### Real-time quantitative polymerase chain reaction (PCR) of telomere length

Genomic DNA (gDNA) was isolated from DFCs with the QIAamp DNA Mini kit (Qiagen). Concentrations were measured with the Nano Drop 2000 (Thermo Scientific). A real-time PCR-based method was used for estimation of telomere length, which was originally described by Gil and Coetzer (2004). The procedure of this method has been previously described in more detail (Morsczeck et al. 2019a).

### PTHrP enzyme-linked immunosorbent assay (ELISA)

The PTHrP ELISA kit was purchased from Aviva Systems Biology (Human PTHLH/ PTHRP ELISA Kit). The kit was used according to the supplier’s protocol. For the analysis of extracellular PTHrP, 100 µl cell culture media supernatant was used.

### Immunofluorescence staining

Cells were fixed in 4% formalin and blocked in 5% normal goat serum and 0.3% Triton X-100 in PBS. A specific human PTHrP rabbit IgG antibody (Abcam #ab224503) and an anti-rabbit IgG Alexa Fluor 488 labeled secondary antibody (Cell Signaling #4412) were used for immunofluorescence according to the manufacturer’s recommendations. Antibodies were diluted in 1% BSA and 0.3% Triton X-100 in PBS. As negative control, cells were incubated only in secondary antibody. The ECLIPSE Ts2-FL inverted microscope (Nikon) with transmitted light or a 470 nm LED lamp and the appropriate epifluorescence filter (excitation filter 440–470 nm, dichronic mirror 500 nm, emission filter 534–555 nm) was used for microscopy. Pictures were obtained with the DS-Fi2 camera (2560 × 1920 pixels, bit depth 24) and DS-L3 control unit (both Nikon). Transmitted light and fluorescent image were overlayed in the software ImageJ 1.52a (National Institutes of Health).

### Statistics

The statistical software SPSS 23 (SPSS Inc., Chicago, IL, USA) was used for one-way ANOVA with Tukey's post hoc tests or alternatively unpaired Student’s *t* test was applied. A *p* value below 0.05 was considered as significant (*).

## Results

**Fig. 1:**
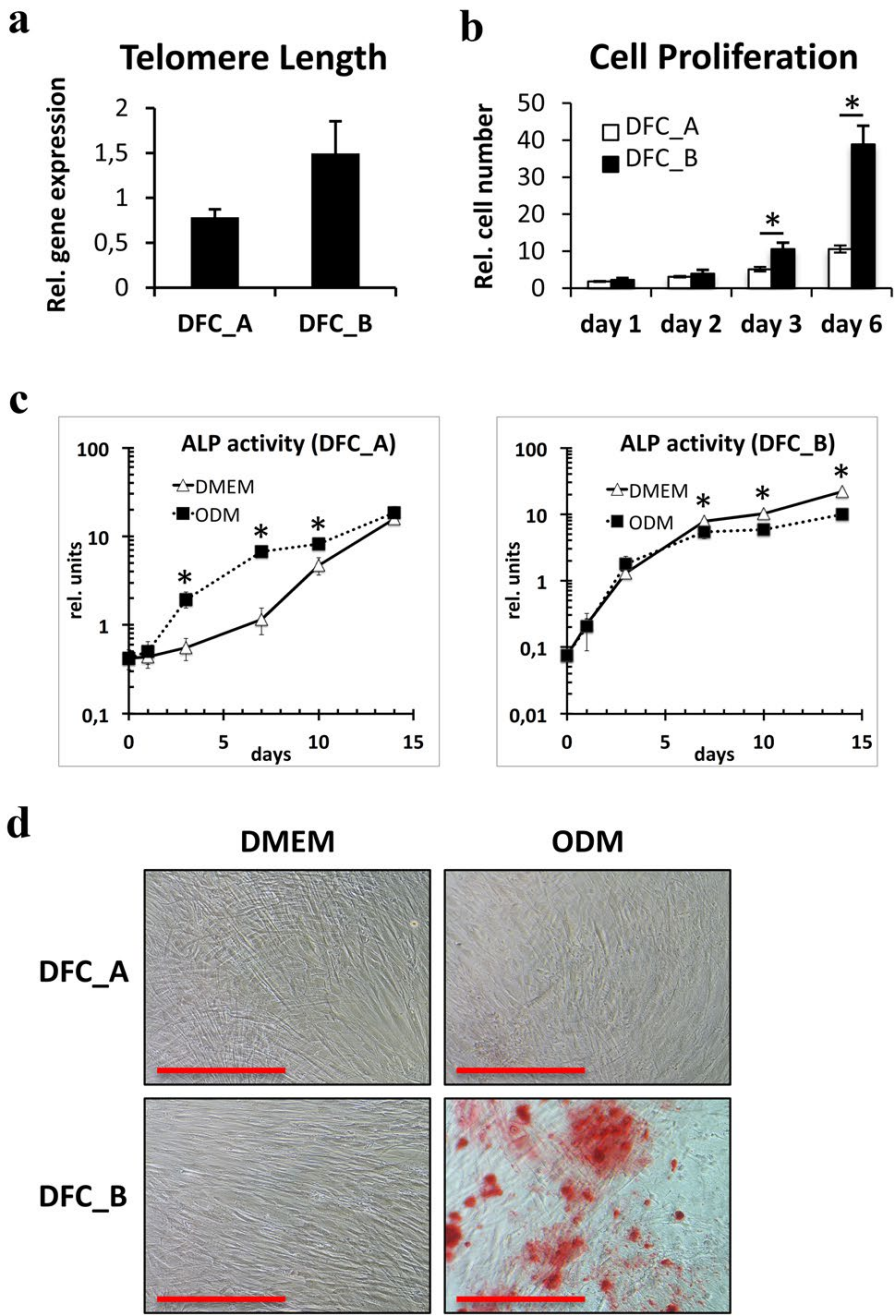
**a** Real-time PCR results for relative telomere length of DFC_A and of DFC_B. Columns represent mean + SD (*n* = 3). **b** Cell numbers of DFC_A and DFC_B were estimated by CCK8 assay at different time points. Columns represent mean + SD (*n* = 8), *p < 0.05. **c** Alkaline phosphatase activity was estimated after the induction of the osteogenic differentiation with dexamethasone (ODM). Control cells were cultivated in standard medium (DMEM). Data points represent mean + SD (*n* = 3), *p < 0.05. **d** Alizarin red staining with DFC_A and DFC_B after 4 weeks long-term cultures in standard medium (DMEM) and osteogenic differentiation medium (ODM). Red bars correspond to 500 µm

We selected for this study two DFC populations, DFC_A and DFC_B, with different cellular characteristics. The telomere length of DFC_B was marginally, but not significantly, longer and the proliferation rate was higher than that of DFC_A (Fig. 1a, b). Moreover, the osteogenic differentiation potentials were different in both cell lines (Fig. 1c, d). While the ALP activity could be induced in DFC_A, mineralization could not be induced after 4 weeks of osteogenic differentiation with a dexamethasone-based medium. Interestingly, the mineralization could be induced with another osteogenic differentiation medium derived from the StemPro™ Osteogenesis Differentiation Kit, but weaker than in DFC_B (data not shown). However, the osteogenic differentiation potential of DFC_B was special. The level of ALP activity of DFC_B rapidly increased both in ODM containing dexamethasone and in standard medium without an osteogenic inducer (Fig. 1c). Although the ALP activity could not be induced with dexamethasone (actually significantly reduced), a strong mineralization was achieved after 4 weeks in dexamethasone-based osteogenic differentiation medium.

**Fig. 2:**
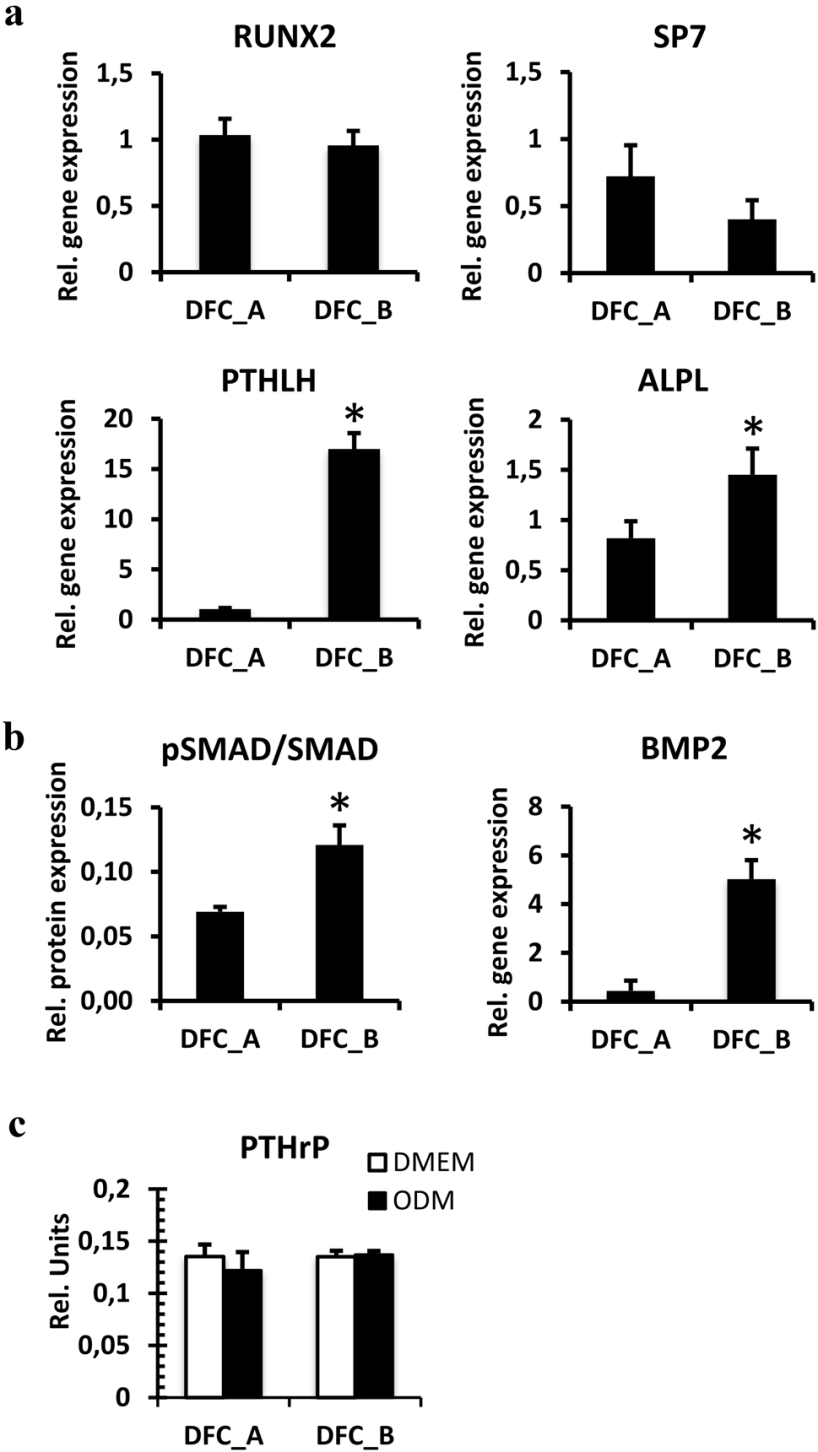
**a** Relative gene expression (real-time RT-PCRs) of osteogenic differentiation markers in DFC_A and in DFC_B. Columns represent mean + SD (*n* = 3), *p < 0.05. **b** Relative degree of the phosphorylation of the SMAD1/5 protein (western blot analyses) and relative gene expression of BMP2 (real-time RT-PCRs) in DFC_A and in DFC_B. Columns represent mean + SD (*n* = 3), *p < 0.05. Whole length blots and a total lane protein image can be found in Supplementary Figure S1. **c** Relative amounts of extracellular PTHrP in cell culture media of DFC_A and DFC_B. DFCs were cultivated in standard medium (DMEM) or in osteogenic differentiation medium (ODM). Columns represent mean + SD (*n* = 3), *p < 0.05

Gene expression of osteogenic differentiation markers such as crucial osteogenic transcription factors RUNX2 and Osterix (SP7) were similarly expressed in both cell lines (Fig. 2a). However, the gene expressions of the gene of ALP (ALPL) and of the PTHrP gene were induced in DFC_B (Fig. 2a). Gene expression of bone morphogenetic protein (BMP2) was also increased in DFC_B (Fig. 2b). Moreover, SMAD1/5 proteins of the BMP pathway are more phosphorylated in DFC_B than in DFC_A (Fig. 2b), which indicated activation of the BMP signaling pathway in DFC_B.

**Fig. 3:**
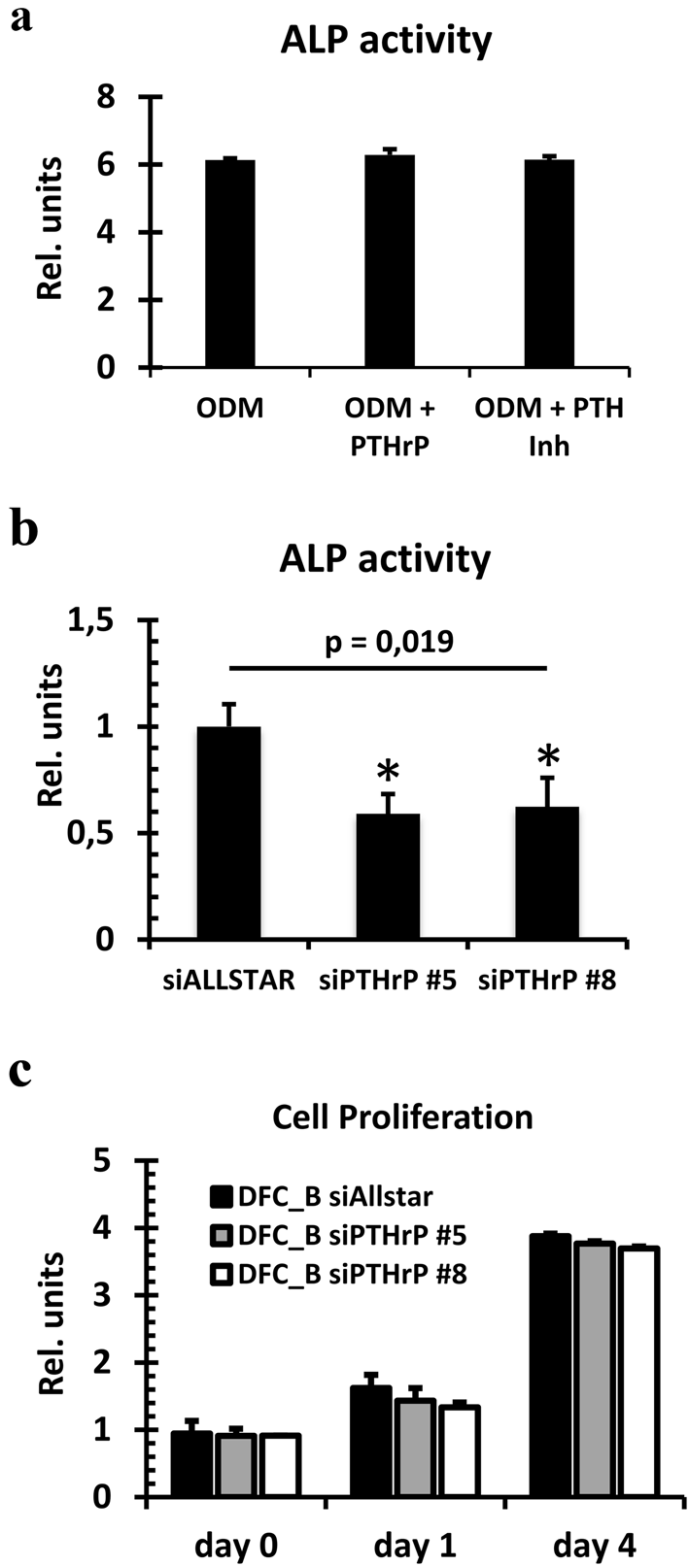
**a** ALP activity in DFC_B after 7 days treatment with PTHrP or with the PTH receptor inhibitor (Inh) PTH (7–34) and osteogenic differentiation. Columns represent mean + SD (*n* = 3). **b** ALP activity in DFC_B after gene silencing of PTHrP with specific siRNAs after 7 days of osteogenic differentiation. For control, an unspecific siRNA (AllStars) was used. Columns represent mean + SD (*n* = 3), *p < 0.05. **c** Estimation of cell proliferation of DFC_B with CCK8 assay after inhibition of PTHrP gene expression. Columns represent mean + SD (*n* = 3)

**Fig. 4:**
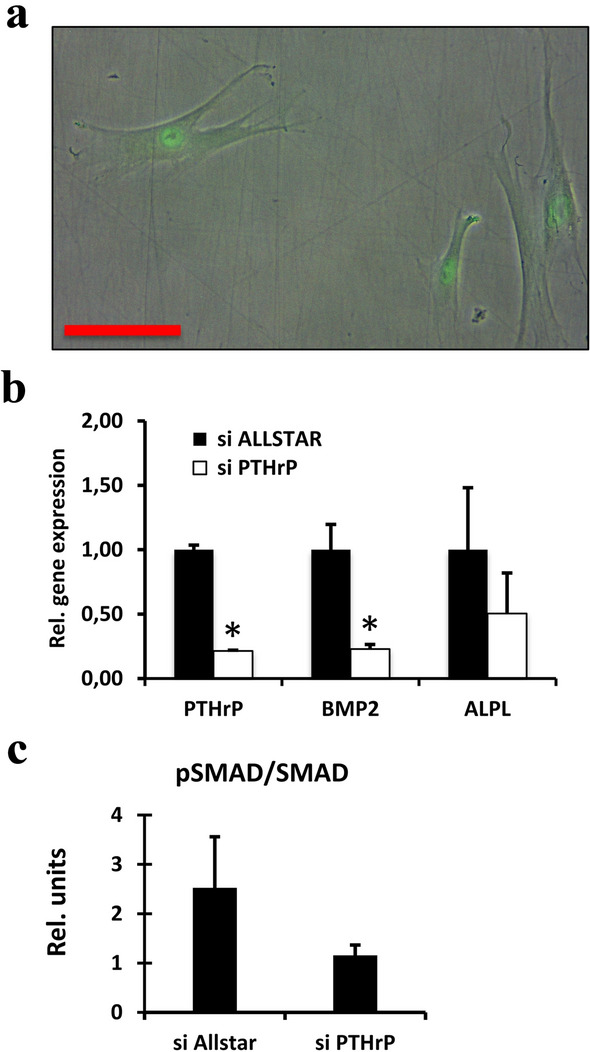
**a** Detection of PTHrP expression in DFC_B by immunofluorescence. Overlay of transmitted light and fluorescence image. The red bar corresponds to 50 µm. Images were obtained with a 40 × magnification objective lens. Exposure time was 0.02 s for transmitted light image and 2 s for fluorescence image. **b** Gene expression (real-time RT-PCRs) of PTHrP (PTHLH), BMP2 and ALP (ALPL) in DFC_B after gene silencing of PTHrP. For control, an unspecific siRNA (AllStars) was used. Columns represent mean + SD (*n* = 3), *p < 0.05. **c** Relative degree of the phosphorylation of the SMAD1/5 protein (western blot analyses) in DFC_B after gene silencing of PTHrP. For control, an unspecific siRNA (AllStars) was used. Columns represent mean + SD (*n* = 3). Whole length blots and a total lane protein image can be found in Supplementary Figure S1

We investigated whether the induced expression of PTHrP is responsible for the better (compared to DFC_A) osteogenic differentiation potential of DFC_B, because a previous study has shown that PTHrP is involved in the expression of ostoegenic differentiation markers such as ALP (Klingelhöffer et al. 2016; Platas et al. 2017). Both cell lines expressed the gene of the parathyroid hormone (PTH)/PTHrP receptor (data not shown). Moreover, DFC_A and DFC_B secreted similar amounts of PTHrP into the extracellular space before and after induction of osteogenic differentiation (Fig. 2c). Interestingly, after 7 days of osteogenic differentiation, the ALP activity could neither be induced nor inhibited by PTHrP or by PTH (7–34), a PTH/PTHrP receptor antagonist (Fig. 3a). To explore whether PTHrP supports the osteogenic differentiation via an intracrine mode, we inhibited the gene expression of PTHrP by specific siRNAs. The ALP activity was inhibited after 7 days of osteogenic differentiation by specific siRNAs for PTHrP (Fig. 3b). However, gene silencing of PTHrP did not influence cell proliferation of DFCs (Fig. 3c). These results suggest that PTHrP supports ALP activity through an intracrine mode that also correlates well with the nuclear expression of PTHrP (Fig. 4a). Moreover, silencing of PTHrP gene expression regulates the gene expression of both BMP2 and ALP (ALPL) (Fig. 4b) and the phosphorylation of the BMP-pathway protein SMAD1/5 (Fig. 4c). PTHrP is therefore likely to be involved in the osteogenic differentiation via the BMP-pathway.

## Discussion

Dental stem cells have already been used in regenerative therapies of many human diseases with frequently superior therapy outcomes and remarkably few adverse effects (Yamada et al. 2020). Besides, products derived from platelet concentrates are considered as promising approach in bone regeneration (Annunziata et al. 2018; De Pascale et al. 2015). However, previous studies have shown diversities between different cell populations, which can be derived from the same tissue (Alraies et al. 2017; Morsczeck et al. 2019a; Prateeptongkum et al. 2016). Furthermore, a previous study has shown that dental stem cells derived even from the same donor have different stem cell characteristics (Prateeptongkum et al. 2016). These differences include important stem cell features such as cell differentiation potentials that are critical for reliable repeatable clinical applications. However, some of these diversities can also be used to learn more about the cellular mechanisms of stem cells. For example, previous studies have shown that induction of cell aging and proliferative and regenerative heterogeneity are associated with different telomere lengths between dental stem cell populations (Alraies et al. 2017; Morsczeck et al. 2019a). Short telomeres are often related to decreased proliferation and stem cell characteristics of MSCs (Al-Qarakhli et al. 2019; Chen et al. 2018). Such comparative studies can be used to screen and isolate high proliferative and multipotent stem cell populations for regenerative medicine. In this context, our study selected two cell populations with different osteogenic differentiation potentials and different proliferation rates.

We found that DFC_B has a superior osteogenic differentiation potential over DFC_A. Our data suggest that the stronger osteogenic differentiation potential of DFC_B is caused by the high endogenous PTHrP expression and the induction of the BMP signaling pathway, which is highly involved in the osteogenic differentiation of DFCs (Kémoun et al. 2007; Morsczeck et al. 2009; Viale-Bouroncle et al. 2012). The gene expression of PTHrP was strongly induced in DFC_B in comparison to the gene expression in DFC_A. However, the concentration of extracellular PTHrP was not induced in DFCs after the induction of osteogenic differentiation. Moreover, the supplementation of the osteogenic differentiation medium with PTHrP did not significantly reduce ALP activity. These results are in contrast to results of our previous study with DFCs (Klingelhöffer et al. 2016). However, the unusually high endogenous ALP activity in DFC_B long-term cultures in standard medium could be an explanation for the absence of PTHrP sensitivity. Furthermore, our results suggest that endogenously expressed PTHrP is preferably localized in the nucleus of DFCs, which was previously also shown in dental follicle cells of murine tooth germs (Zhang et al. 2019b).

We further found that DFC_B has a higher proliferation rate in combination with longer telomeres compared to DFC_A. This correlation is compliant with the findings in the literature (Al-Qarakhli et al. 2019; Chen et al. 2018). Furthermore, short telomeres are correlated with the onset of cellular senescence and subsequently reduced differentiation potential (Li et al. 2017; Morsczeck et al. 2019b). It is unclear to which extent the telomere length and proliferation rate are responsible for the observed differences in osteogenic differentiation potential. However, since the main factor found for the cell population differences in this work is the endogenous expression of PTHrP, and the cell proliferation was not affected by siRNA knockdown of the gene for PTHrP, we conclude that at least the observed effect on osteogenic differentiation potential caused by PTHrP is not directly dependent on proliferation rate and telomere length.

While previous studies indicated that PTHrP peptides promote tooth eruption and inhibit the osteogenesis of dental follicle cells (Klingelhöffer et al. 2016; Zhang et al. 2019b), our new data suggest that PTHrP supports the expression of BMP2 and the alkaline phosphatase activity, and both are strongly involved in the osteogenic differentiation of DFCs. This conclusion is in line with other studies about PTHrP and the osteogenic differentiation. Here, different moieties of PTHrP were associated with the protection of osteoblastic cells against cellular stress (Ardura et al. 2017; Platas et al. 2017; Portal-Núñez et al. 2018). Moreover, the nuclear localized PTHrP inhibits cellular senescence, regulates DNA damage checkpoints and induces the expression of anti-oxidative enzymes, which is directly associated with the regulation of the DNA damage checkpoint pathway (Fiaschi-Taesch et al. 2009; Miao et al. 2008; Zhang et al. 2018).

We suppose that the role of PTHrP in DFC_B is different from the PTHrP-PPR (PTH/PTHrP receptor) autocrine signaling pathway (Nagata et al. 2020; Takahashi et al. 2019). Here, PTHrP is secreted into the extracellular space and its binding to PPR regulates the cementoblastic differentiation of DFCs. An interruption of the PPR leads to the unphysiological formation of cementum with loss of tooth attachment to the alveolar bone. Nagata et al. suggested that this pathway is highly important for both the formation of the periodontal attachment apparatus and tooth eruption (Nagata et al. 2020). We believe that during tooth development and osteogenic differentiation, PTHrP plays several (including conflicting) roles in DFCs. However, our study did not include genome-wide or broad pathway scanning analyses and only focused on distinct molecular markers and pathways. Thus, we were unable to finally discover the diversity of PTHrP functions in DFCs. With the use of next-generation sequencing or microarray platforms, additional studies could further explore this diversity and provide a basis for uncovering possible connections between the different functions.

## Electronic supplementary material

Below is the link to the electronic supplementary material.

Supplementary file1 (DOCX 13 kb)

## References

[CR1] Abzhanov A, Rodda SJ, McMahon AP, Tabin CJ (2007). Regulation of skeletogenic differentiation in cranial dermal bone. Development (Cambridge, England).

[CR2] Al-Qarakhli AMA, Yusop N, Waddington RJ, Moseley R (2019). Effects of high glucose conditions on the expansion and differentiation capabilities of mesenchymal stromal cells derived from rat endosteal niche. BMC Mol Cell Biol.

[CR3] Alraies A, Alaidaroos NYA, Waddington RJ, Moseley R, Sloan AJ (2017). Variation in human dental pulp stem cell ageing profiles reflect contrasting proliferative and regenerative capabilities. BMC Cell Biol.

[CR4] Annunziata M, Guida L, Nastri L, Piccirillo A, Sommese L, Napoli C (2018). The role of autologous platelet concentrates in alveolar socket preservation: a systematic review. Transfusion medicine and hemotherapy : offizielles Organ der Deutschen Gesellschaft fur Transfusionsmedizin und Immunhamatologie.

[CR5] Ardura JA, Portal-Núñez S, Castelbón-Calvo I, Martínez de Toda I, de La Fuente M, Esbrit P (2017). Parathyroid hormone-related protein protects osteoblastic cells from oxidative stress by activation of MKP1 phosphatase. J Cell Physiol.

[CR6] Chen L, Xia W, Hou M (2018). Mesenchymal stem cells attenuate doxorubicin-induced cellular senescence through the VEGF/Notch/TGF-β signaling pathway in H9c2 cardiomyocytes. Int J Mol Med.

[CR7] De Pascale MR, Sommese L, Casamassimi A, Napoli C (2015). Platelet derivatives in regenerative medicine: an update. Transfus Med Rev.

[CR8] Di Bernardo G, Galderisi U, Fiorito C, Squillaro T, Cito L, Cipollaro M, Giordano A, Napoli C (2010). Dual role of parathyroid hormone in endothelial progenitor cells and marrow stromal mesenchymal stem cells. J Cell Physiol.

[CR9] Fiaschi-Taesch N, Sicari B, Ubriani K, Cozar-Castellano I, Takane KK, Stewart AF (2009). Mutant parathyroid hormone-related protein, devoid of the nuclear localization signal, markedly inhibits arterial smooth muscle cell cycle and neointima formation by coordinate up-regulation of p15Ink4b and p27kip1. Endocrinology.

[CR10] Gil ME, Coetzer TL (2004). Real-time quantitative PCR of telomere length. Mol Biotechnol.

[CR11] Kémoun P, Laurencin-Dalicieux S, Rue J, Farges J-C, Gennero I, Conte-Auriol F, Briand-Mesange F, Gadelorge M, Arzate H, Narayanan AS, Brunel G, Salles J-P (2007). Human dental follicle cells acquire cementoblast features under stimulation by BMP-2/-7 and enamel matrix derivatives (EMD) in vitro. Cell Tissue Res.

[CR12] Klingelhöffer C, Reck A, Ettl T, Morsczeck C (2016) The parathyroid hormone-related protein is secreted during the osteogenic differentiation of human dental follicle cells and inhibits the alkaline phosphatase activity and the expression of DLX3. Tissue Cell 48(4):334–339 10.1016/j.tice.2016.05.007 27368119

[CR13] Li C, Wei G-J, Xu L, Rong J-S, Tao S-Q, Wang Y-S (2017). The involvement of senescence induced by the telomere shortness in the decline of osteogenic differentiation in BMSCs. Eur Rev Med Pharmacol Sci.

[CR14] Miao D, Su H, He B, Gao J, Xia Q, Zhu M, Gu Z, Goltzman D, Karaplis AC (2008). Severe growth retardation and early lethality in mice lacking the nuclear localization sequence and C-terminus of PTH-related protein. Proc Natl Acad Sci USA.

[CR15] Morsczeck C (2006). Gene expression of runx2, Osterix, c-fos, DLX-3, DLX-5, and MSX-2 in dental follicle cells during osteogenic differentiation in vitro. Calcif Tissue Int.

[CR16] Morsczeck C (2015). Molecular mechanisms in dental follicle precursor cells during the osteogenic differentiation. Histol Histopathol.

[CR17] Morsczeck C, Reichert TE (2017). The dexamethasone induced osteogenic differentiation of dental follicle cells. Histol Histopathol.

[CR18] Morsczeck C, Reichert TE (2018). Dental stem cells in tooth regeneration and repair in the future. Exp Opin Biol Ther.

[CR19] Morsczeck C, Moehl C, Götz W, Heredia A, Schäffer TE, Eckstein N, Sippel C, Hoffmann KH (2005). In vitro differentiation of human dental follicle cells with dexamethasone and insulin. Cell Biol Int.

[CR20] Morsczeck C, Schmalz G, Reichert TE, Völlner F, Saugspier M, Viale-Bouroncle S, Driemel O (2009). Gene expression profiles of dental follicle cells before and after osteogenic differentiation in vitro. Clin Oral Invest.

[CR21] Morsczeck C, Reck A, Reichert TE (2019). Short telomeres correlate with a strong induction of cellular senescence in human dental follicle cells. BMC Mol Cell Biol.

[CR22] Morsczeck C, Reck A, Reichert TE (2019). WNT5A supports viability of senescent human dental follicle cells. Mol Cell Biochem.

[CR23] Nagata M, Ono N, Ono W (2020). Mesenchymal progenitor regulation of tooth eruption: a view from PTHrP. J Dent Res.

[CR24] Platas J, Guillén MI, Gomar F, Castejón MA, Esbrit P, Alcaraz MJ (2017). Anti-senescence and Anti-inflammatory Effects of the C-terminal Moiety of PTHrP Peptides in OA Osteoblasts. J Gerontol Series A Biol Sci Med Sci.

[CR25] Portal-Núñez S, Ardura JA, Lozano D, Martínez de Toda I, de La FuenteHerrero-Beaumont MG, Largo R, Esbrit P (2018). Parathyroid hormone-related protein exhibits antioxidant features in osteoblastic cells through its N-terminal and osteostatin domains. Bone Jt Res.

[CR26] Prateeptongkum E, Klingelhöffer C, Müller S, Ettl T, Morsczeck C (2016). Characterization of progenitor cells and stem cells from the periodontal ligament tissue derived from a single person. J Periodontal Res.

[CR27] Schiano C, Soricelli A, De Nigris F, Napoli C (2019). New challenges in integrated diagnosis by imaging and osteo-immunology in bone lesions. Exp Rev Clin Immunol.

[CR28] Sonoyama W, Liu Y, Fang D, Yamaza T, Seo B-M, Zhang C, Liu H, Gronthos S, Wang C-Y, Wang S, Shi S (2006). Mesenchymal stem cell-mediated functional tooth regeneration in swine. PLoS ONE.

[CR29] Sybil D, Jain V, Mohanty S, Husain SA (2020). Oral stem cells in intraoral bone formation. J Oral Biosci.

[CR30] Takahashi A, Nagata M, Gupta A, Matsushita Y, Yamaguchi T, Mizuhashi K, Maki K, Ruellas AC, Cevidanes LS, Kronenberg HM, Ono N, Ono W (2019). Autocrine regulation of mesenchymal progenitor cell fates orchestrates tooth eruption. Proc Natl Acad Sci USA.

[CR31] Viale-Bouroncle S, Felthaus O, Schmalz G, Brockhoff G, Reichert TE, Morsczeck C (2012). The transcription factor DLX3 regulates the osteogenic differentiation of human dental follicle precursor cells. Stem Cells Dev.

[CR32] Vortkamp A (2000). The Indian hedgehog–PTHrP system in bone development. Ernst Scher Res Found Workshop.

[CR33] Winer J, Jung CK, Shackel I, Williams PM (1999). Development and validation of real-time quantitative reverse transcriptase-polymerase chain reaction for monitoring gene expression in cardiac myocytes in vitro. Anal Biochem.

[CR34] Yamada Y, Nakamura-Yamada S, Konoki R, Baba S (2020). Promising advances in clinical trials of dental tissue-derived cell-based regenerative medicine. Stem Cell Res Ther.

[CR35] Zhang Y, Chen G, Gu Z, Sun H, Karaplis A, Goltzman D, Miao D (2018). DNA damage checkpoint pathway modulates the regulation of skeletal growth and osteoblastic bone formation by parathyroid hormone-related peptide. Int J Biol Sci.

[CR36] Zhang J, Ding H, Liu X, Sheng Y, Liu X, Jiang C (2019). Dental follicle stem cells: tissue engineering and immunomodulation. Stem Cells Dev.

[CR37] Zhang J, Liao L, Li Y, Xu Y, Guo W, Tian W, Zou S (2019). Parathyroid hormone-related peptide (1–34) promotes tooth eruption and inhibits osteogenesis of dental follicle cells during tooth development. J Cell Physiol.

[CR38] Zhou T, Pan J, Wu P, Huang R, Du W, Zhou Y, Wan M, Fan Y, Xu X, Zhou X, Zheng L, Zhou X (2019). Dental follicle cells: roles in development and beyond. Stem Cells Int.

