# Supplementary material for: High endogenous expression of parathyroid hormone-related protein (PTHrP) supports osteogenic differentiation in human dental follicle cells
Source: Histochem Cell Biol. 2020 Jul 24;154(4):397–403. doi: 10.1007/s00418-020-01904-7 (PMC8616871; doi:10.1007/s00418-020-01904-7)
Supplement: Supplementary file 1 — Supplementary file1 (DOCX 13 kb) [file 418_2020_1904_MOESM1_ESM.docx]

Supplementary Figure Legend:

**Fig. S1** (**a**) Whole length Western Blots of phosphorylated and unphosphorylated SMAD1/5 proteins in DFC_A and in DFC_B and total lane protein image. Blots correspond to the pSMAD/SMAD ratio in Fig. 2b (left). (**b**) Whole length Western Blots of phosphorylated and unphosphorylated SMAD1/5 proteins in DFC_B after gene silencing of PTHrP and total lane protein image. For control an unspecific siRNA (AllStars) was used. Blots correspond to the pSMAD/SMAD ratio in Fig. 4c.
